# The effect of a calcium soap and jackfruit leaf extract on blood metabolites, oxidative stress biomarkers and follicle growth of crossbreed doelings

**DOI:** 10.5713/ab.25.0337

**Published:** 2025-08-25

**Authors:** Yusti Pujiawati, I Komang Gede Wiryawan, Lilis Khotijah, Mohamad Agus Setiadi, Simon Petrus Ginting

**Affiliations:** 1 Graduate School of Nutrition and Feed Science, Faculty of Animal Science, Bogor Agricultural University, Bogor, Indonesia; 2 Research Center for Animal Husbandry, Research Organization for Agriculture and Food, National Research and Innovation Agency of Indonesia, Cibinong Science Center, Bogor, Indonesia; 3 Department of Nutrition and Feed Technology, Faculty of Animal Science, Bogor Agricultural University, Bogor, Indonesia; 4 Division of Reproduction and Obstetrics, Department of Veterinary Clinic, Reproduction and Pathology, Faculty of Veterinary Medicine, Bogor Agricultural University, Bogor, Indonesia

**Keywords:** Antioxidants, Blood Metabolites, Follicle Development, Hormones, Polyunsaturated Fatty Acid

## Abstract

**Objective:**

This study evaluates the effect of a flushing ration containing polyunsaturated fatty acids (PUFA) and jackfruit leaf extracts (JLEs) on the follicle growth, reproductive hormone profiles and oxidative stress state of doelings.

**Methods:**

Fifteen primiparous Boer×Jawarandu doelings (23.21±3.57 kg body weight, 10–11 months old) were divided into three groups: control (PUFA flushing ration), control+JLE (200 mg/kg dry matter intake [DMI]), and control+Ca-soap (calcium soap)+JLE (200 mg/kg DMI). The following parameters were measured: production performances, follicle development, blood metabolites, antioxidant status, estradiol, and progesterone concentration.

**Results:**

The doelings’ glucose levels showed significant differences (p<0.05) during the post-flushing phase, on days 19, and day 21 after estrus. An increase in glucose level was observed following the administration of the control+Ca-soap+JLE ration. However, on day 21 after estrus, the highest glucose level was detected in the control+JLE treatment group (p<0.05). In addition, the doelings’ cholesterol levels were elevated on days 19 and 21 with the administration of the control+JLE ration. These changes in glucose and cholesterol levels between phases are presumed to be associated with the increased demand for hormone synthesis and follicular development. JLE supplementation reduced malondialdehyde (MDA) levels across all phases (p<0.05), indicating lower oxidative stress. Moreover the administration of the control+JLE flushing ration resulted in the highest estradiol hormone level on day 19 after estrus compared to other treatments. This finding is consistent with the greater number of medium-sized follicles observed in doelings receiving the same treatment. Additionally, including JLE in the flushing ration also resulted in increased doelings’ progesterone hormone levels during the luteal phase.

**Conclusion:**

JLE can mitigate oxidative stress by reducing blood MDA levels during the pre-mating phase. Follicle formation, especially of larger and medium-sized follicles, may be accelerated when a flushing proportion of PUFA is combined with JLE.

## INTRODUCTION

Synthetic estradiol has been widely used in managing reproductive disorders in livestock, such as anestrus. Altough the negative impacts of this hormone on food safety have not been scientifically confirmed, its use has been increasingly restricted in several countries, including Australia, Canada, South Africa, and Japan. Moreover, the European Union has banned the import of livestock products treated with this hormone [1]. These regulations are driven by concerns regarding hormonal residues in food products. Consequently, efforts to reduce the use of synthetic hormones continue to be encouraged. Goats are a vital livestock resouce in marginal areas of Asia. In Indonesia goat populations outnumber sheep, reflecting their strategic role as a source of source of food and income for rural communities [2]. According to the Observatory of Economic Complexity [3], in 2022, Indonesia exported live goats valued at USD 33,600, primarily to Malaysia. Thus, the abundant availability of local goats and growing export market opportunities offer significant potential to increase export value. Mediterranean countries, the European Union, and Latin America represent promising export destinations [4]. However, to realize expanding this export potential, Indonesian goat breeders must comply with the strict regulatory standards of destination countries, including restrictions on the use of synthetic hormones. A crossbreeding program using Boer males and Jawarandu females was conducted to improve reproductive performance. The program revealed that prolificacy is highly influenced by hormonal balance, which affects the development of dominant follicles and ovulation. A previous study also found that improving the nutritional status of goats through flushing diets containing 1.0 to 1.5 times the maintenance nutritional requirements can enhance oocyte quality [5].

Reproductive diets for goat are typically supplemented with oils rich in long-chain polyunsaturated fatty acids (PUFA). PUFA as an energy source and plays a role in follicular development, acts as a precursor for reproductive hormones and twinning rates [6,7]. However, excessive intake of PUFA can impair rumen microbial activity and digestion. Biohydrogenation by rumen microbes converts PUFA into saturated fatty acids to reduce toxicity, decreasing PUFA availability for ovarian function. This process can be mitigated through saponification using alkali and calcium salts (Table 1) [8].

Moreover, previous studies reported that increased energy demands correlate with higher free radical production during the reproductive period. An imbalance between oxidative stress and antioxidant capacity adversely affects oocyte development, induces granulosa cell apoptosis, and leads to corpus luteum dysfunction [9,10]. Enhancing antioxidant capacity can be achieved through supplementation with plant secondary metabolites [11]. These leaves contain active compounds, including chlorogenic acid, quercetin, rutin, and kaempferol [12]. Ethanolic extracts of jackfruit leaves demonstrate antioxidant activity of 56.50 ppm [13]. An *in vivo* study also showed that jackfruit leaf extract (JLE) can reduced oxidative stress in diabetic rats [14].

In this study, the researchers analyzed the goats’ blood metabolite levels to evaluate the effect of the flushing ration on energy availability required for follicular development and as a precursor for reproductive hormone synthesis. The levels of malondialdehyde (MDA), superoxide dismutase (SOD), and catalase (CAT) were assessed to determine the antioxidant effects of jackfruit leaf on lipid peroxidation. Reproductive function during the pre-mating period was evaluated based on the availability of reproductive hormones, namely estradiol and progesterone, as well as ovarian follicular development.

## MATERIALS AND METHODS

### Jackfruit leaf extract and calcium soap preparation

The researcher used mature jackfruit leaves, specifically the fourth and fifth leaves from the tip of the branch. The collected leaves were dried at 50°C until completely dry and then ground using a blender to obtain a powder (80 mesh). The maceration method, using 70% ethanol as the solvent with at a leaf powder-to-solvent ratio of 1:2 to 1:2.5 (w/v), was employed to obtain the extract. The samples were incubated in an airtight container for 24 hours with occasional stirring, followed by filtration using a coarse filter cloth. The maceration process was repeated twice. The resulting filtrate was concentrated using a rotary evaporator with a water bath temperature of 40°C to obtain a thick extract. Metabolite analysis was conducted using the Vanquish UHPLC system coupled with a Q Exactive Plus Orbitrap High-Resolution Mass Spectrometer (HRMS) (Thermo Fisher Scientific), with an Accucore C18 column (100×2.1 mm, 1.5 μm; Thermo Fisher Scientific). The metabolite profiling results are shown in Table 2.

Calcium soap was prepared using the double decomposition method employed by Jenkins and Palmquist [15]. Sunflower seed oil and flaxseed oil were heated to 80°C and then mixed with sodium hydroxide (NaOH). The mixture was stirred until it thickened, after which calcium chloride (CaCl_2_) was added to induce solidification.

### Animals, diet, and experimental design

The study was conducted at Animal Nutrition and Feed Laboratory of Bogor Agricultural University, Indonesia. The research was conducted from June to April 2023. The study’s subjects comprised fifteen (n = 15) primiparous Boer× Jawarandu crossbreed doelings (average body weight [BW] of 23.21±3.57 kg), aged 10–11 months old, with a body condition score (BCS) of <2.0 (1–5 scales). The doelings were randomly allocated into three groups and five replications. The first group was given a flushing ration containing PUFA (control). The second group was given a flushing ration control and JLE 200 mg/kg dry matter intake (DMI) (control+JLE). The third group was given a flushing ration with calcium soap and JLE 200 mg/kg DMI (control+Ca-soap+JLE). The doelings were individually housed in pens measuring 1.5 m^2^, each equipped with a diet trough and water container. The subjects’ nutritional requirements were determined based on the standards set by the NRC [16]. The doelings were fed three times a day at 08:00 a.m, 12:00 p.m and 03:00 p.m., and drinking water was provided *ad libitum*. Diet quantities were adjusted every two weeks based on a DMI requirement of 3.5% of BW.

The composition and nutrient content of the diet ingredients are presented in Table 3. The extract was premixed with a small amount of concentrate to ensure homogeneity and then orally administered before the morning feeding. A 14 day adaptation period to the new diet was conducted. The doeling’s BW and BCS were measured every two weeks using a digital scale and visual observation, following the method described by Koyuncu and Altınçekiç [17].

All doelings involved in the study had never undergone estrus synchronization. Estrus synchronization was performed following a 14-day flushing period using the double injection prostaglandin method. Each goat received 0.6 mL of Lutaprost (equivalent to 0.263 mg of cloprostenol sodium) with an 11-day interval between injection.

### Blood sampling

**Figure 1:**
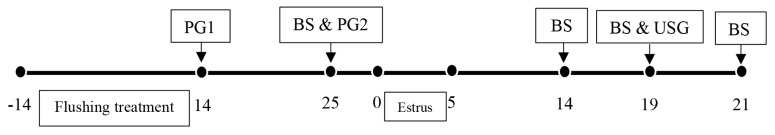
Protocol of the experiment from flushing, estrus synchronization, blood sample collection, and ultrasonography. BS, blood sampling; PG, prostaglandin F2α injection; USG, ultrasonographic scanning.

Blood samples (5 mL each) were obtained via the jugular vein of all doelings and were stored in plain vacutainer tubes and anticoagulant vacutainer tubes. Each sample was centrifuged at 700×g for 15 minutes, then transferred to 1.5 mL microcentrifuge tubesand stored at −20°C in a freezer. The blood samples were collected before the morning feeding and at specific time points: at day 14 post flushing (PF14), day 14 after the first estrus, day 19 after the first estrus and day 21 after the first estrus (Figure 1).

### Measurement of hormones blood levels, metabolites, and antioxidant indicators

The doelings’ estradiol concentrations were measured at two phases: day 19 and day 21 after estrus. The estradiol levels were analyzed using the enzyme-linked immunosorbent assay (ELISA) method with a commercial goat-specific estradiol kit (MBS702565; Mybiosource). Progesterone was measured on day 14 after estrus using the ELISA method with a commercial goat-specific progesterone kit (EA0005Go; BT Lab).

Blood parameters including glucose, cholesterol, and non-esterified fatty acids (NEFA) were measured in four phases. Glucose concentrations were analyzed using the glucose kit (112191 AKL 20101803460), and cholesterol levels were determined using the cholesterol kit (101592, AKL 10101803466). Both kits were manufactured in Germany and distributed by Rajawali Nusindo. NEFA concentrations were measured using the colorimetric assay kit (E-BC-K013-S; Elabscience).

Body antioxidant capacity was also assessed in four phases. MDA levels were determined using the thiobarbituric acid method with the Elabscience kit (E-BC-K025). SOD activity was measured using the T-SOD activity assay kit (E-BC-K019-2; Elabscience), and CAT activity was determined using the CAT activity assay kit (E-BC-K031-S; Elabscience).

### Follicular development

The number and diameters of follicles, as well as the diameters of the uterus, ovaries, and corpus luteum, were measured on day 17 after the first estrus using transrectal ultrasonography with a 7.5 MHz probe (Aloka SSD-500, SN M07265; Aloka). The diameters of the follicles were determined by measuring the two furthest points of the respective structures. Follicles were categorized based on their diameters into three groups: small follicles (2–3 mm), medium-sized follicles (4–5 mm), and large follicles (>5 mm) [18].

### Statistical analysis

The data were analyzed using analysis of variance (ANOVA) and Duncan’s multiple range test, with statistically significant differences determined at p*<*0.05 using IBM SPSS Statistics 29. The ANOVA and Duncan’s tests were employed to evaluate the effect of the treatments on the doelings’ production performances, estradiol and progesterone hormone levels, and follicle counts. Meanwhile, the researchers used two-way ANOVA, followed by Tukey’s post hoc test for the multiple comparisons of blood metabolite parameters and oxidative stress markersThe subsequent model was applied to evaluate the differences among treatments:


(1) Yij=μ+αi+eij

Where Yij is dependent variable, μ represents overall mean, αi is the fixed effect for supplementation of JLE, and eij is the experimental error assumed to be NID with (0, 2e).

## RESULTS

### Nutrient intake, average daily gain and body condition score

The results indicate that the intake of DM, crude protein (CP), total digestible nutrient (TDN), ether extract (EE) and NFE were not affected by the flushing ration and JLE supplementation (p>0.05). Flushing ration did not affect DMI because the CP and TDN contents were formulated to be isoenergetic and isonitrogenous across all treatments. DMI ranged from 29 to 32 g/kg BW, while CP intake was approximately 6 g/kg BW, and TDN intake ranged from 21 to 22 g/kg BW. Meanwhile, the intake of linoleic acid (LA), α-linolenic acid (ALA), and Ca were affected in the flushing rations groups (p<0.05). The intake of LA and ALA in the control+Ca-soap+JLE treatment was lower compared to the other treatments. However, this treatment also showed an increase in Ca intake. The variation in LA, ALA and Ca intake can be attributed to the lower levels of LA and ALA, and the highest level of Ca in the control+Ca-soap+JLE, compare to the control and control+JLE groups.

The doelings’ BCS during the flushing phase was not affected by the flushing rations treatments (p>0.05; Table 4). The final BCS ranged from 2.00 to 2.30 (Table 4). The results also indicated that the doelings’ BWgain and final BW were also not affected by the flushing ration treatments (p>0.05).

### Plasma glucose, cholesterol, non esterified fatty acid

Two-Way ANOVA test results revealed a significant relationship between the flushing ration and the observation phase on blood glucose levels (p<0.05; Table 5). This finding suggests that blood glucose concentration was influenced by both the dietary treatment and the phase of observation. On day 19 post-estrus, the group receiving the control+Ca-soap+JLE diet showed a significantly higher blood glucose level compared to the other treatment groups at the same time phase (p<0.05). However, on day 21, the glucose level in this group decreased significantly to 56.14±4.37 mg/dL.

Next, the doelings’ blood cholesterol levels exhibited a fluctuating pattern influenced by the interaction between the flushing diet treatments and observation phases (p<0.05; Table 5). A significant increase in cholesterol concentration was observed on day 21 post-estrus in the control+JLE group, which was higher than in the other dietary treatment groups. The control+Ca-soap+JLE group showed consistently lower cholesterol levels up to day 19 post-estrus, although an increase was noted on day 21 (136.59±35.00 mg/dL).

However, no significant interaction was found between the flushing diet treatments and the observation phases on NEFA levels (p>0.05). Overall, the control+JLE group showed significantly higher NEFA concentrations compared to both the control group and the control+Ca-soap+JLE group (p<0.05). In addition, NEFA levels were significantly affected by the observation phase (p<0.05), with the highest concentration recorded on day 19 post-estrus compared to other phases.

### Oxidative stress indicators (malondialdehyde, superoxide dismutase, and catalase)

The MDA, T-SOD, and CAT analyses of flushing rations supplemented with JLE are presented in Table 6. A significant interaction was observed between the flushing ration and the observation phase on MDA levels (p<0.05), indicating that the effect of the flushing ration on lipid peroxidation varied depending on the observation phase. Based on the statistical analysis, no interaction was found between the flushing ration treatment and the observation phase on T-SOD activity (p>0.05). However, the observation phase had significant effect on T-SOD activity (p<0.05), with a gradual increase observed on days 14 and 21 post-estrus. The lowest T-SOD activity was recorded on PF14 (16.45 U/mL), while the highest activity was observed on day 21 post-estrus (41.75 U/mL). CAT enzyme activity in the blood showed a significant difference across the flushing ration and the observation phases (p<0.05). The highest activity was recorded at PF14, which significantly decreased on days 19 and 21 post estrus. This indicates a reduction in endogenous antioxidant capacity during the late post-estrus phase. The control+JLE group showed significantly higher CAT activity compared to both the control group and the control+Ca-soap+JLE group (p<0.05).

### Serum estradiol and progesterone hormone levels

The doelings’ serum estradiol levels measured at day 19 post-estrus and day 21 post-estrus are presented in Table 7. The control+JLE group had higher serum estradiol levels compared to the control and control+Ca-soap+JLE treatments (p<0.05). However, estradiol levels on day 21 post-estrus did not differ significantly between treatment groups (p>0.05). Serum progesterone hormone were analyzed at day 14 post-estrus. The goat received JLE had higher serum progesterone levels compared to the control group (p<0.05).

### Number and diameters of the follicles

The number of follicles and follicle diameter in doeling fed a diet containing PUFA and JLE are presented in Table 8. The results indicate that the number of medium and large follicles was affected by flushing rationand JLE (p<0.05). Medium follicles were more frequently found in the control+JLE group, with a value of 2.60. Large follicles were more frequently found in the control+Ca-soap+JLE group, with a value of 2.80 (Table 8). Descriptively, the diameter of follicle in doeling fed a flushing diet containing PUFA and supplemented with JLE showed that diameter of small follicles ranged from 2.92 to 3.21 mm, medium follicles ranged from 4.28 to 4.50 mm, and large follicles ranged from 6.07 to 6.38 mm.

## DISCUSSION

Nutrient intake is a key factor influencing the reproductive performance of goats. The flushing ration and JLE did not affect feed intake or palatability. The intake of dry matter (DM), CP, and TDN met the nutritional requirements of doelings in the breeding period with a BW of 23 kg, namely 26.5 g/kg BW, 1.8 g/kg BW, and 14.0 g/kg BW [16]. In addition, the EE content in the diet did not affect DMI, as the EE level remained below the 6% threshold [19].

The saponification process of oils resulted in a reduction of LA and ALA concentrations in the control+Ca-soap+JLE diet. This reduction was attributed to the lower percentage of oil used following the addition of alkali and calcium salts. Thus, the LA and ALA content in the Ca-soap decreased by approximately 40%–50% compared to the pure oils. As a result, the intake of LA and ALA in the control+Ca-soap+JLE group was lower than in the other dietary treatments. Nevertheless, the LA intake in this group still met the NRC [16] requirement for a 23 kg doeling, which is 0.577 g/head/day.

The use of CaCl_2_ in the production of Ca-soap also increased the Ca content in the control+Ca-soap+JLE diet. This led to an increase in Ca intake, reaching 1.66% of DMI. The increase intake of Ca may have a positive impact on follicular development. Which is consistent with this study’s finding of a higher number of large follicles in the Ca-soap treatment group. The female reproductive system requires Ca^2+^ as a regulatory second messenger in oocyte maturation mechanisms [20]. Other studies have also shown that Ca^2+^ deficiency can impair oocyte maturation and reduce the number of somatic cells during bovine embryo transfer procedures [21].

Next, the final BCS of 2.0–2.3 observed in this study is still considered within the ideal category, as reported by Widiyono et al [22], who stated that a BCS of 2–3 is optimal for stimulating ovarian activity, increasing the number of large follicles, and supporting corpus luteum development. However, the BCS values in this study fall on the lower end of the ideal range, despite the average daily gain (ADG) reaching 140–150 g/head/day. The high ADG without a corresponding increase in BCS suggests that the weight gain was primarily distributed to non-fat tissues such as muscle and body fluids.

This study monitored the doelings’ blood metabolite dynamics at several phases during the estrus cycle. The results showed that glucose concentration was significantly affected by the interaction between the flushing ration and the estrus cycle. The control+Ca-soap+JLE diet stimulated an increase in glucose concentration during PF and on day 19 post-estrus. Thus, an increase in glucose concentration prior to estrus plays a crucial role in stimulating the secretion of gonadotropin-releasing hormon, which subsequently regulates the secretion of follicle-stimulating hormone and luteinizing hormone to support follicular maturation [23]. The saponification of PUFAs into Ca-soap in the flushing ration increase the availability of glucose precursors. This process reduces the acetate-to-propionate ratio in the rumen, where propionate serves as the main glucose precursor through the gluconeogenic pathway [24]. Similarly, El-Nour et al [25] reported an increase in blood glucose concentration following Ca-soap supplementation. The catechin content in JLE is also presumed to contribute to the reducing of the acetate-to-propionate ratio and, enhancing the availability of glucose precursors. This finding is consistent with Aemiro et al [26], who reported that supplementation with 50 g/kg DM of Sunphenon 30S-O (containing 10.25 g catechins) reduced the acetate-to-propionate ratio by 4.3%. However, the effect of Ca-soap on glucose levels in this study still requires further validation as there was no treatment group that a received a flushing diet with Ca-soap without the addition of JLE.

Moreover, the plasma glucose concentration in doelings fed the control+Ca-soap+JLE diet decreased significantly on day 21 post-estrus. This decline is attributed to the metabolic differences between day 19 and 21 post-estrus, resulting from hormonal changes. At the time of estrus (day 21), glucose levels decreased concurrently with an increase in blood cholesterol levels. A contrasting pattern was observed on day 19 in the same group, where glucose concentration increased while cholesterol concentration decreased. This shift in metabolic response is likely influenced by the body’s demand for cholesterol during estrus, which is required for estrogen synthesis. This explanation aligns with the sharp rise in estradiol levels observed on day 21 (Table 7). Cholesterol synthesis depends on the availability of acetyl-CoA, which is produced during glucose glycolysis. This process is believed to contribute to the reduction in glucose levels, which occurs in parallel with the increase in cholesterol concentration on day 21 in the control +Ca-soap+JLE group. Additionally, enhanced glycolysis may be promoted by the antidiabetic effects of catechins present in JLE. Catechins have been reported to improve insulin sensitivity, facilitating cellular glucose uptake [27].

Furthermore, the increased demand for cholesterol and glucose on days 19 and 21 post-estrus may have been met through the mobilization of body fat, as indicated by elevated NEFA concentrations. The highest NEFA level was observed in the control+JLE group, which may be due to the inhibition of cholesterol synthesis by flavonoid compounds present in JLE, resulting in a greater reliance on fat mobilization to meet cholesterol needs. Flavonoids are known to activate sterol regulatory element-binding protein 2 (SREBP-2), which in turn upregulates the low-density lipoprotein receptor (LDLR) gene expression, thereby reducing blood cholesterol levels [28]. This findings aligns with Sugimoto-Kawabata et al [29], who reported an increase in labeled NEFA components incorporated into cholesterol in rats administered cholesterol-lowering agents.

MDA concentration reflects the level of lipid peroxidation in the body, which is positively correlated with oxidative stress. The highest MDA concentration was recorded in the PF phase, followed by a sharp decline through day 21 post-estrus. This pattern indicates a reduction in lipid peroxidation and oxidative stress risk, likely due to adaptive physiological responses. Notably, the decline in MDA levels was more pronounced in goats supplemented with JLE, suggesting that JLE effectively inhibited excessive lipid peroxidation. Jackfruit leaves have been identified to contain catechin, which possesses hydroxyl groups with strong affinity for metal ions that act as catalysts in oxidative reactions [30].

This study also found that T-SOD activity was influenced by the phase of the estrous cycle. This condition coincided with increased concentrations of glucose, cholesterol, and NEFA concentrations during the same phase. Estrus and ovulation are physiological processes that require high energy to support the secretion of reproductive hormone. This elevated metabolic activity contributes to free radical production, which is counterbalanced by increased T-SOD activity to maintain redox homeostasis [31].

CAT, another endogenous antioxidant enzyme, was influenced by both the flushing ration and the estrous cycle stage. CAT levels were higher during the PF phase and on day 14 post-estrus, but declined on days 19 and 21 post-estrus. In this study, the CAT concentration pattern did not align with T-SOD activity. The discrepancy in their activity patterns may be due to the compensatory role of glutathione peroxidase (GPx), which also neutralizes H_2_O_2_ and inhibits lipid peroxidation. Although GPx activity was not measured in this study, the low MDA levels on days 19 and 21 suggest possible involvement of GPx activity. Additionaly, CAT concentrations were higher in doelings fed JLE-supplemented diets compared to the control flushing ration. This finding is consistent with a meta-analysis by Lucio-Ruíz et al [32], which reported that flavonoid supplementation significantly enhanced antioxidant enzyme activity, including CAT, in small ruminants. CAT levels were also significantly increased in cells pre-treated with catechin (Epigallocatechin Gallate, EGCG) prior to oxalate exposure [33], an effect mediated via the Nuclear Factor Erythroid 2-related factor 2 (Nrf2) activation pathway [34].

Reproductive hormone secretion also plays a critical role in regulating the estrous cycle in doelings. The control+JLE diet stimulated higher serum estradiol secretion on day 19 post-estrus. This increased estradiol level was supported by higher cholesterol availability during the same phase. The unprotected LA and ALA components in the control+JLE diet were more effective at stimulating cholesterol synthesis than the Ca-soap-protected fats. This observation is supported by Meza-Villalvazo et al [35] who reported that unprotected corn oil supplementation elevated both cholesterol and estradiol concentrations in sheep. In addition, the nicotiflorin content in JLE (Table 2) may have also contributed to increased estradiol secretion. A previous study found that supplementation with *Cnidoscolus acanthus* leaf extract at 100 mg/kg, which contains nicotiflorin as a major metabolite, was shown to increase serum estradiol levels in female rats [36]. Progesterone secretion was also higher in the JLE-supplemented group compared to the control group. The flavonoid compounds in JLE may have indirectly stimulated progesterone production by reducing lipid peroxidation. This explanation is constent with the lower MDA levels and increased CAT activity observed in doelings receiving JLE-containing diets. Moreover, meta-analytical evidence suggests that flavonoid supplementation supports corpus luteum function by inhibiting lipid membrane peroxidation, thus maintaining progesterone secretion [37].

**Figure 2:**
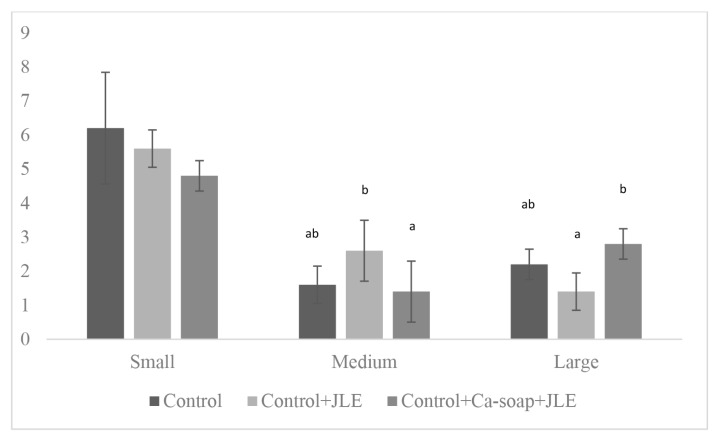
Bar chart with error bars showing the effect of flushing diet and JLE on the number of follicles. Error bars represent standard deviation ^a,b^ Means in the same row with different superscripts differ significantly at p<0.05. JLE, jackfruit leaf extract.

Finally, the consumption of flushing rations supplemented with JLE increased the number of medium and large follicles (Figure 2). The medium follicles observed in the control+JLE treatment group were positively correlated with elevated estradiol concentrations on day 19 post-estrus. Since estradiol is produced by granulosa cells of developing follicles, follicle number is positively associated with estradiol levels. Large follicles were more commonly found in doelings fed Ca-soap and JLE diets. The higher calcium intake from Ca-soap was beneficial to follicle maturation. Meanwhile, disruption in Ca^2+^ signaling has been shown to impair oocyte maturation, disturb meiosis and fertilization [20]. Thus the control+Ca-soap+JLE ration fed during the premating period stimulated glucose concentration, stabilized cholesterol levels, and reduced the risk of oxidative stress, supporting estradiol secretion and follicular development.

## CONCLUSION

JLE can mitigate oxidative stress by reducing blood MDA levels and improve CAT activity during the pre-mating period. The combination of a PUFA flushing ration with JLE may enhance follicular development, particularly medium and large-sized follicles.

## Figures and Tables

**Table 1:** Compositions of the fatty acids (%) of sunflower oil, flaxseed oil, Ca-soap sunflower oil and Ca-soap flaxseed oil

Fatty acid	Sunflower oil	Flaxseed oil	Ca-soap sunflower oil	Ca-soap flaxseed oil
Linolenic acid	0.3268	49.94405	0.1521	22.68695
Linoleic acid	58.07595	16.7396	26.67085	8.93535
Oleic acid	31.1675	21.9072	16.25175	14.041
Omega 6 fatty acid	58.07595	16.7396	26.67085	8.93535
Omega 3 fatty acid	1.0507	50.12275	0.1521	22.7044
Polyunsaturated fatty acid	59.13845	66.8821	26.8314	31.65265
Unsaturated fatty acid	90.5954	89.39925	43.54365	46.64265
Omega 9 fatty acid	31.1675	21.9072	16.25175	14.0505
Monounsaturated fatty acid	31.45685	22.51715	16.71225	14.98995
Saturated fatty acid	9.2054	10.48165	4.9507	5.44135

**Table 2:** Identification of secondary metabolites in Jackfruit leaf extract (JLE) by LC-MS/MS

Compound group	Compound name	Formula	RT	MW
Flavonoid	Catechin	C15 H14 O6	7.499	290.07957
Hydroxy fatty acid	(15Z)-9,12,13-Trihydroxy-15-octadecenoic acid	C18 H34 O5	13.208	330.24116
Phenolic acid	5-O-methyl embelin	C18 H28 O4	14.933	308.19923
Phenolic acid	Chlorogenic acid	C16 H18 O9	18.818	294.21992
Alkaloid	Cinchonain Ia	C24 H20 O9	10.601	452.1107
Hyroxycarboxylic acid	D-(−)-Quinic acid	C7 H12 O6	1.237	192.06289
Flavonoid	Glabridin	C20 H20 O4	18.973	324.1365
Flavonoid	Nicotiflorin	C27 H30 O15	8.195	594.15974
Flavonoid	Ostruthin	C19 H22 O3	17.907	298.16054
Chlorophyll catabolite	Pheophorbide A	C35 H36 N4 O5	26.702	592.26936

Structure image retrieved from ChemSpider ( www.chemspider.com ).

LC-MS/MS, liquid chromatography–tandem mass spectrometry; RT, retention time; MW, molecular weight.

**Table 3:** Ingredients and nutrient contents of ration (DM%) fed to the experimental goats

Variables	Group		

	Control	Control+JLE	Control+Ca-soap+JLE
Ingredients			
Napier grass	30.00	30.00	30.00
Cassava pulp	15.00	15.00	14.03
Coconut meal	20.00	20.00	20.00
Soybean meal	10.00	10.00	10.00
Pollard	16.61	16.61	16.61
Molases	4.00	4.00	4.00
Sunflower oil (SO)	1.42	1.42	-
Flaxseed oil (FO)	0.87	0.87	-
Ca-soap SO	-	-	2.01
Ca-soap FO	-	-	1.25
CaCO_3_	0.70	0.70	0.70
Premix*	0.70	0.70	0.70
Salt	0.70	0.70	0.70
Jackfruit leaf extracts	-	200 mg/kg DMI	200 mg/kg DMI
Nutrient contents (%DM)			
CP	19.67	19.67	19.65
EE	4.12	4.12	3.42
CF	18.35	18.35	18.11
TDN	71.53	71.53	70.08
Ca	0.70	0.70	1.67
P	0.51	0.51	0.51
LA	1.00	1.00	0.68
ALA	0.47	0.47	0.32
LA:ALA ratio	2.13	2.13	2.14

Control = flushing diet without supplementation of jackfruit leaf extract, control+JLE = flushing diet with addition 200 mg/kg DMI jackfruit leaf extract, control+Ca-soap+JLE = flushing diet contained Ca-soap and addition 200 mg/kg DMI jackfruit leaf extract.

DM, dry matter; JLE, jackfruit leaf extract; DMI, dry matter intake; CP, crude protein; EE, ether extract; CF, crude fiber; TDN, total digestible nutrient; Ca, calcium; P, phosphorus; LA, linoleic acid; ALA, linolenic acid.

**Table 4:** Nutrien intake, body condition score (BCS) and average daily gain of Boer×Jawarandu does fed a flushing diet with PUFA and jackfruit leaf extract (JLE)

Variable (g/head/day)	Group		

	Control	Control+JLE	Control+Ca-soap+JLE
DMI	701.56±100.40	683.57±133.57	736.72±131.84
Crude protein	138.00±19.75	134.46±26.27	144.77±25.91
TDN	501.83±71.81	488.96±95.54	516.29±92.39
Crude fiber	128.74±18.42	125.44±24.51	133.42±23.88
Ether extract	28.90±4.13	28.16±5.50	25.20±4.51
NFE	343.41±49.15	334.61±65.39	355.76±63.66
Calcium	4.91±0.70^a^	4.78±0.93^a^	12.30±2.20^b^
Phosporus	3.58±0.51	3.49±0.68	3.76±0.67
LA	7.02±1.01^b^	6.84±1.34^b^	5.01±0.90^a^
ALA	3.30±0.47^b^	3.21±0.63^b^	2.36±0.42^a^
Initial BCS	1.60±0.55	1.60±0.42	1.80±0.27
Final BCS	2.00±0.67	2.20±0.61	2.30±0.57
Intial live weigth (kg)	23.12±3.82	22.39±4.23	23.49±3.42
Final live weight (kg)	27.39±4.30	27.18±4.76	27.88±4.35
Average daily gain (g/day)	142.33±28.18	159.73±31.69	146.33±34.87

a,b Means in the same row with different superscripts differ significantly at p<0.05.

DMI, dry matter intake; TDN, total digestible nutrient; NFE, nitrogen free extract; LA, linoleic acid; ALA, linolenic acid.

**Table 5:** Plasma glucose, cholesterol, non esterified fatty acid (NEFA) concentration in boer×jawarandu does fed a flushing diet with PUFA and jackfruit leaf extract (JLE)

Variable	Group			Mean±SD

	Control	Control+JLE	Control+Ca-soap+JLE	
Glucose (mg/dL)				
PF14	34.83±6.39^A^	40.67±5.73^AB^	49.40±8.97^ABC^	42.12±9.05^a^
Day 14	70.89±9.64^DE^	74.56±5.23^EF^	69.08±8.94^DE^	71.21±8.08^bc^
Day 19	76.12±6.42^EF^	63.69±5.87^CDE^	88.23±2.15^F^	77.05±10.81^c^
Day 21	64.67±3.25^CDE^	72.96±7.66^DEF^	56.14±4.37^BCD^	65.36±8.56^b^
Mean±SD	62.95±17.23	60.66±16.13	65.29±16.91	
Cholesterol (mg/dL)				
PF14	75.75±17.14^AB^	67.54±5.45^AB^	60.58±7.26^A^	67.99±12.56^a^
Day 14	86.49±1.89^AB^	99.78±10.68^ABC^	81.06±19.11^AB^	88.85±13.05^b^
Day 19	88.98±10.06^AB^	87.35±10.08^AB^	62.99±12.20^A^	79.12±16.02^ab^
Day 21	110.97±5.26^BC^	164.72±41.19^D^	139.71±32.48^CD^	136.59±35.00^c^
Mean±SD	90.86±16.90^ab^	104.07±42.67^b^	86.64±39.20^a^	
NEFA (mmol/L)				
PF14	0.024±0.001	0.050±0.025	0.035±0.018	0.036±0.019^a^
Day 14	0.053±0.007	0.075±0.040	0.044±0.015	0.057±0.026^ab^
Day 19	0.045±0.006	0.112±0.010	0.059±0.015	0.072±0.032^b^
Day 21	0.067±0.032	0.068±0.023	0.049±0.026	0.061±0.025^ab^
Mean±SD	0.047±0.022^a^	0.076±0.033^b^	0.047±0.019^a^	

A–F, a–c Means not sharing the same subscripts were different at a = 0.05 significant level. The ^A–F^ Subscripts distinguished interactive differences and the ^a^ and ^b^ subscripts distinguished the main effect.

SD, standard deviation; PF, post-flushing.

**Table 6:** Malondialdehid (MDA), superoxide dismutase (T-SOD) and catalase (CAT) concentration in boer×jawarandu does fed a flushing diet with PUFA and jackfruit leaf extract (JLE)

Variable	Group			Mean±SD

	Control	Control+JLE	Control+Ca-soap+JLE	
MDA (nmol/L)				
PF14	86.66±29.30^C^	58.33±15.00^BC^	34.45±19.32^AB^	59.81±29.61^b^
Day 14	18.50±6.66^A^	10.98±2.70^A^	7.64±2.45^A^	12.37±6.13^a^
Day 19	28.71±4.74^AB^	0.80±0.81^A^	3.68±1.81^A^	11.06±13.54^a^
Day 21	26.55±6.73^AB^	4.71±1.29^A^	4.66±2.94^A^	11.97±11.55^a^
Mean±SD	40.11±31.31^b^	18.71±25.06^a^	12.61±15.71^a^	
T-SOD (U/mL)				
PF14	12.93±2.24	20.65±7.12	15.77±3.82	16.45±5.39^a^
Day 14	27.59±2.23	29.17±5.48	24.75±12.06	27.17±6.99^b^
Day 19	30.90±1.45	26.64±4.47	30.74±1.64	29.43±3.25^b^
Day 21	45.34±3.26	40.66±4.67	39.24±6.69	41.75±5.19^c^
Mean±SD	29.19±12.20	29.28±8.93	27.63±10.85	
CAT (U/mL)				
PF14	12.53±3.67	19.22±5.40	14.73±2.41	15.49±4.56^b^
Day 14	12.21±0.89	13.01±6.12	17.35±8.31	14.19±5.71^b^
Day 19	3.21±0.93	4.77±4.31	2.60±2.37	3.53±2.68^a^
Day 21	4.35±0.36	11.29±0.56	5.18±0.57	6.94±3.31^a^
Mean±SD	8.08±4.81^a^	12.07±6.67^b^	9.97±7.54^ab^	

A–C, a–c Means not sharing the same subscripts were different at a = 0.05 significant level. The ^A–C^ subscripts distinguished interactive differences and the ^a^ and ^b^ subscripts distinguished the main effect.

PUFA, polyunsaturated fatty acid; SD, standard deviation; PF, post-flushing.

**Table 7:** Serum estradiol concentration in boer×jawarandu does fed a flushing diet with PUFA and jackfruit leaf extract (JLE)

Variable (ng/mL)	Groups		

	Control	Control+JLE	Control+Ca-soap+JLE
Estradiol			
Day 19 post estrus	36.87±9.37^a^	233.33±97.24^b^	32.70±2.67^a^
Day 21 post estrus	333.03±103.28	229.25±121.35	276.17±95.44
Progesteron	5.37±1.54^a^	17.64±6.34^b^	17.73±4.48^b^

a,b Means in the same row with different superscripts differ significantly at p<0.05.

PUFA, polyunsaturated fatty acid.

**Table 8:** Numbers and diameters of the follicles in boer×jawarandu does fed diet flushing supplemented with jackfruit leaf extract (JLE)

Variables	Group		

	Control	Control+JLE	Control+Ca-soap+JLE
Number of follicle			
Small	6.20±1.64	5.60±0.55	4.80±0.45
Medium	1.60±0.55^ab^	2.60±0.90^b^	1.40±0.90^a^
Large	2.20±0.84^ab^	1.40±0.55^a^	2.80±0.45^b^
Total follicle	10.00±2.12	9.60±1.40	9.00±0.71
Small	2.94 (n = 31)	2.92 (n = 28)	3.21 (n = 24)
Medium	4.28 (n = 8)	4.39 (n = 13)	4.50 (n = 7)
Large	6.38 (n = 11)	6.26 (n = 7)	6.07 (n = 14)

a,b Means in the same row with different superscripts differ significantly at p<0.05.
